# Mapping Uncharted Lead-Free Halide Perovskites and Related Low-Dimensional Structures

**DOI:** 10.3390/ma17020491

**Published:** 2024-01-19

**Authors:** Anna Dávid, Julia Morát, Mengyun Chen, Feng Gao, Mats Fahlman, Xianjie Liu

**Affiliations:** 1 Laboratory of Organic Electronics (LOE), Department of Science and Technology, Linköping University, 60174 Norrköping, Sweden; mats.fahlman@liu.se; 2 Department of Physics, Chemistry and Biology (IFM), Linköping University, 58183 Linköping, Sweden; julia.morat@liu.se (J.M.); mengyun.chen@liu.se (M.C.); feng.gao@liu.se (F.G.)

**Keywords:** lead-free halide (double) perovskites, low-dimensional structures, crystal structure, synthesis

## Abstract

Research on perovskites has grown exponentially in the past decade due to the potential of methyl ammonium lead iodide in photovoltaics. Although these devices have achieved remarkable and competitive power conversion efficiency, concerns have been raised regarding the toxicity of lead and its impact on scaling up the technology. Eliminating lead while conserving the performance of photovoltaic devices is a great challenge. To achieve this goal, the research has been expanded to thousands of compounds with similar or loosely related crystal structures and compositions. Some materials are “re-discovered”, and some are yet unexplored, but predictions suggest that their potential applications may go beyond photovoltaics, for example, spintronics, photodetection, photocatalysis, and many other areas. This short review aims to present the classification, some current mapping strategies, and advances of lead-free halide double perovskites, their derivatives, lead-free perovskitoid, and low-dimensional related crystals.

## 1. Introduction: Synthetic Lead-Free Halide Perovskites and Related Low-Dimensional Structures

**Figure 1:**
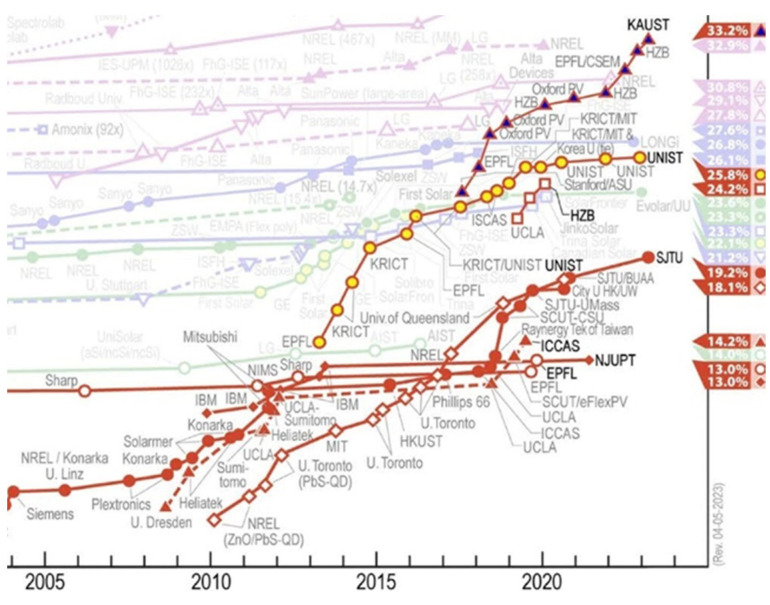
Cell efficiency chart by the US National Renewable Energy Laboratory in May 2023 [4]. This plot is courtesy of the National Renewable Energy Laboratory, Golden, CO, USA.

The invention of crystalline silicon solar cells was a milestone of energy conversion technology development in the 1950s. This achievement led to extensive research and accelerating progress in device fabrication within the photovoltaic field. Now, more than half a century later, several solutions are available [1]. One branch of these devices employs versatile synthetic metal halide perovskites in different architectures, for instance, the Grätzel cell [2,3]. The word perovskite is an umbrella term encompassing both all-inorganic and hybrid organic–inorganic compounds with a perovskite crystal structure. The incorporation of perovskites in dye-sensitized solar (Grätzel) cells catalyzed perovskite research, and remarkable efficiency has been reached with those absorbers. For instance, in 2023, the research group of Prof. De Wolf announced a certified power conversion efficiency (PCE) of 33.2% for monolithic perovskite/silicon tandem solar cell construction. The US National Renewable Energy Laboratory is publishing a constantly updated chart where one can follow the efficiency of devices developed over the timeline (see Figure 1) [4].

In general, perovskite research has found that materials with perovskite crystal structures may also be of interest to several other optoelectronic applications due to their relatively easy processability and unique properties. Other than photovoltaics, examples from the constantly growing list of application areas include photodetectors, spintronic devices, light-emitting diodes, and photocatalysts [5,6]. The most prominent representative compounds from the group of perovskites are lead-based, for instance, methylammonium lead iodide. Lead is abundant, cheap, and easily recyclable from several sources. From a techno-economic aspect, these materials may be competitive in markets such as the Internet of Things and building-integrated photovoltaics due to their high optical absorption coefficient, remarkable defect tolerance, and high electronic dimensionality [7]; moreover, facile film preparation and advanced techniques make in situ and operando studies upon perovskite-based devices feasible to significantly push the development of this field [8]. One of the main issues with these materials is the toxicity of lead, which makes large-scale production challenging from the point of sustainability and safety [9,10,11,12]. The daily intake of lead should not exceed 490 μg for an adult, according to the Food and Agricultural Organization/World Health Organization, but there are no guidelines for children. If lead enters the human body, it is distributed through red blood cells and imposes its toxicity and may cause the symptoms listed in Table 1 [13]. However, in the case of methylammonium lead iodide perovskite, precursor PbI_2_ only accounts for 3.68% of the global human toxicity potential, while methylammonium iodide has a contribution of 62.31% according to the Life Cycle Assessment by Zhang et al. [14].

**Figure 2:**
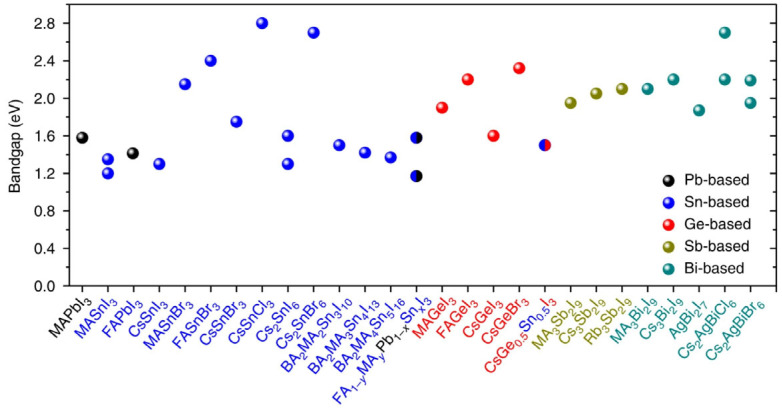
Bandgaps of several lead-free perovskites [17] compared to most studied lead-based ones. Reproduced with permission under the terms of the Creative Commons CC BY license.

Great efforts have been taken to substitute lead in the halide perovskite crystal lattice, but no substitute has shown as remarkable performance in photovoltaics as pristine ones so far. The substitution of lead with other Group IVA elements like tin (or germanium) is an obvious approach; however, perovskites formed with Sn^2+^ are facing stability issues due to their oxidation from +2 to the toxicologically inactive +4 state [15,16]. Figure 2 shows some examples of lead-free perovskites and the most studied lead-based perovskites from the viewpoint of their bandgaps, which is one crucial factor of absorber materials for developing photovoltaic devices [17]. One must not ignore the fact that the suggested compositions contain elements that are either scarce, reliant on heavy metal production, or toxic [9].

**Figure 3:**
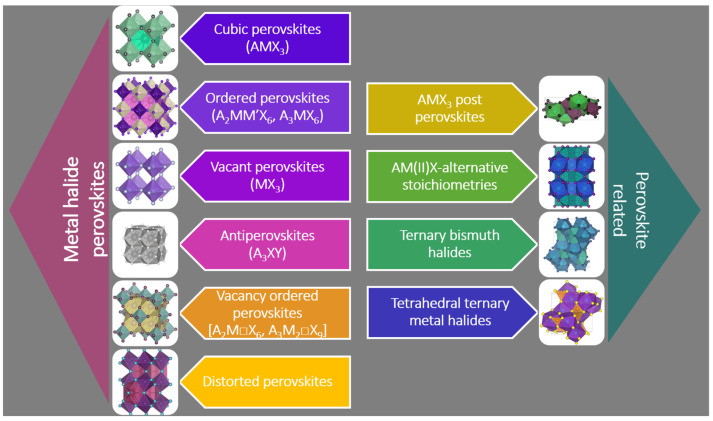
Classification and structures of perovskite and perovskite-related unit cell structures, where A is an organic/inorganic cation, M is a metal cation, X is a halide anion, Y is chalcogenide, and □ is a vacancy.

The attempt to find lead-free materials with as good properties as the lead-containing perovskites led to a vast urge to explore the rich perovskite field which covers thousands of compounds demonstrating major potential in, for instance, energy storage devices [18] and optoelectronic [19,20], spintronic [21], and photocatalytic [5] applications. Perovskites are a great branch of materials, and the name denotes a certain crystal structure with an AMX_3_ stoichiometry, which is composed of MX_6_ corner-sharing octahedra bordering A-site cations. This review focuses on a subgroup of them, halide perovskite, and related low-dimensional lattices which comprise halide (X) octahedra that surround a positively charged metal ion (M). There are examples of M being an organic monovalent cation [22]. In the interstices of these octahedra, another positive ion (A) can be found. Constituent ion A can be either an inorganic ion, for instance, cesium, or a small organic cation like methyl ammonium ion; however, up to now, no systems with an organic–inorganic mixture for this site [5] have been reported. These building blocks can form several different dimensionalities and symmetries dependent on the constituting or vacant ions’ radii [23], valences, synthetic technique, additives, presence of chiral and racemic ligands, and external conditions [24] (see examples in Figure 3 based on the classification by Akkerman and Mana [25]). Akkerman and Mana presented an overview of the group’s breadth and complexity, classifying the representatives by their crystal structure, and identifying six subgroups [25].

Figure 3 shows not only numerous examples of perovskite structures but also solids that are not built up by the network of corner sharing octahedra due to a lack of stability or because of additives that cause derivation from an originally stable lattice. If the structure involves edge and/or face-sharing octahedra MX_6_, the literature may name them “perovskitoid” [26]. The research field of low-dimensional structures is closely related to the area of perovskites; one example to illustrate this is their application as scaffolds for perovskite growth for effective surface defect passivation in perovskite solar cells [23,26].

In this short review, we will generally concentrate on exploring lesser-researched lead-free structures and compositions in the perovskite field and related areas. Since tin halide perovskites and vacancy-ordered structures are highly researched as lead-free perovskites, their discussion is outside of the scope of this work, but a comprehensive review was recently published about those branches [27]. Our goal is to help in the rational material design process by sharing recent advancements in fully inorganic lead-free halide double perovskites and lower dimensionality hybrid perovskite-related or derived structures.

## 2. Extract of Roadmaps on Inorganic Lead-Free Halide Double Perovskites: Versatility, Properties, Development, and Challenges

### 2.1. Mapping the Stability of Halide Double Perovskites

**Figure 4:**
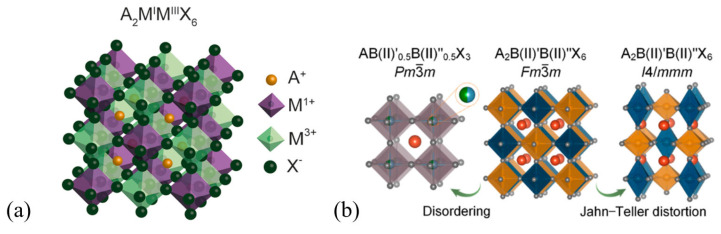
(**a**) Rock-salt-ordered DP structure [32]. Copyright © 2018 American Chemical Society. This publication is licensed under CC-BY-NC-ND. (**b**) Phase transitions from the rock-salt-ordered DP to simple perovskite and Jahn–Teller distorted double perovskite [29]. Copyright © 2022 American Chemical Society.

One way to eliminate divalent lead from the crystal structure is to replace it with a merger of ions with an average charge of +2. This is the case in double perovskites (DPs) (elpasolites), in which this substitution occurs via having a + 1 and a + 3 charged central cation in halide octahedra alternating through the crystal structure, expressed by the A_2_MM′X_6_ fixed stoichiometric formula (Figure 4a shows the crystal structure, Figure 4b marks ion M with B) [28]. There are examples of achieving stable rock-salt-ordered DP structures where both M and M′ are divalent, but usually, these components rather form the stoichiometry of a simple perovskite with a disordered configuration, AM(II)M′(II)X_3_, due to the zero charge difference, as shown in Figure 4b [29]. Having a different order than the rock-salt arrangement by a couple of compositions with A_2_M(I)M(II)X_5_ stoichiometry has also been reported [30]. Vacancy-ordered A_2_M(IV)X_6_ DPs are not discussed here in detail [31].

There are thousands of combinations of elements that may result in DP stoichiometry; thus, screening them is quite a challenge. Despite the vast number of possible combinations, up until 2023, “only” around 350 halide DPs had been synthesized. Theoretical calculations suggest that around 600 further compounds may be synthesized since many of the combinations from the list of thousands are unstable [33].

In the perovskite field, the crystallographic stability of compounds is usually predicted by three tolerance factors that depend on the oxidation state of ion A (n_A_) and the ionic radii of A, M, M′, and X ions (r_A_, r_M_, and r_X_, respectively, where r_M_ is the arithmetic mean of the two M-site radii in the DP lattices). These numbers are usually referred to the following names in the literature and can be calculated using the following equations:

- Goldschmidt tolerance factor:

$$t=\frac{\left(r_{A}+r_{X}\right)}{\sqrt{2}\left(r_{M}+r_{X}\right)}$$

- Octahedral factor:

$$\mu =\frac{r_{M}}{r_{X}}$$

- Tolerance factor by Bartel et al. [34]:

$$\tau =\frac{r_{X}}{r_{M}}-n_{A}\left(n_{A}-\frac{\frac{r_{A}}{r_{M}}}{ln\left(\frac{r_{A}}{r_{M}}\right)}\right)$$

**Figure 5:**
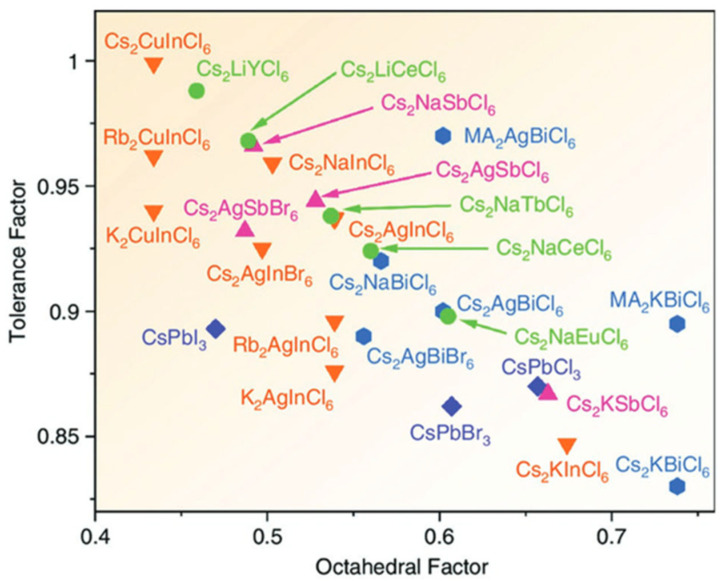
Space of octahedral and tolerance factor values of some halide DPs [35] © 2020 Wiley-VCH GmbH.

To form the perovskite crystal structure, the following criteria must be met: Goldschmidt factor between 0.81 and 1.11, octahedral factor between 0.41 and 0.9, and τ below 4.18 to form a perovskite crystal structure [33,34]. Figure 5 shows the position of several well-known perovskites in the octahedral factor–tolerance factor space, indicating their crystallographic stability.

**Figure 6:**
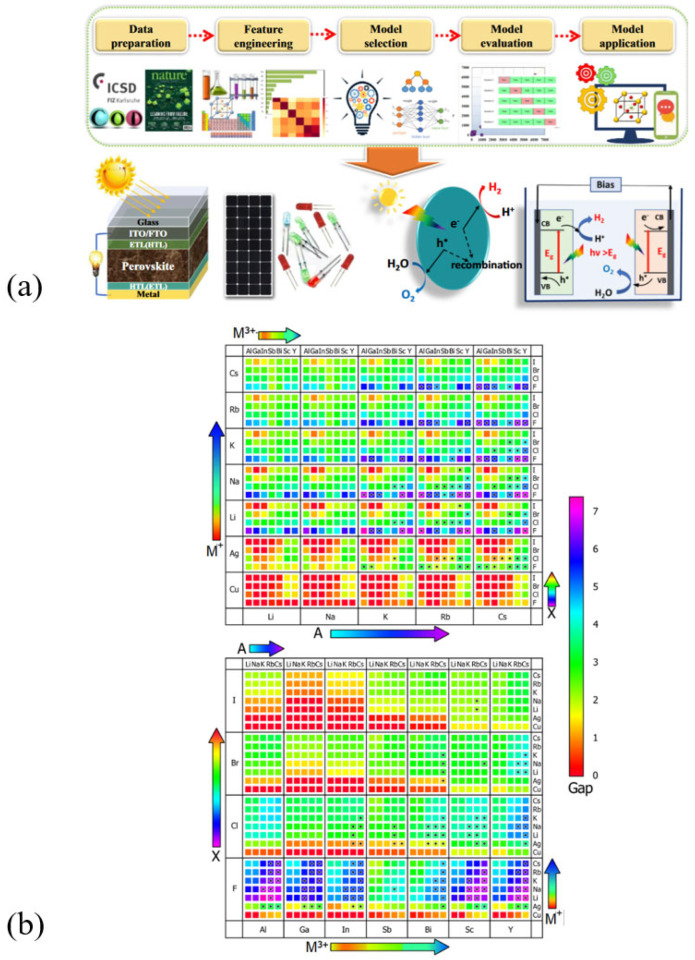
(**a**) General workflow machine learning in perovskite and related applications [40]. Reproduced with permission under the terms of the Creative Commons CC BY license. (**b**) Map of GGA-calculated bandgaps of 980 DB; the black plus signs indicate thermodynamically stable structures [38]. Copyright © 2020, American Chemical Society.

In rare instances, predictions based solely on geometrical factors can be inaccurate. One example is Cs_2_CuBiCl_6_, which is unstable but meets the crystallographic criteria. These cases suggest that it is necessary to analyze the thermodynamic processes involved in the formation and decomposition reactions [36]. These compounds can break down into binary, ternary, or quaternary salts. If the decomposition reactions are poorly understood, there may be contradictions regarding stability. To aid research and development in the field of materials science, an open-access database was created under the name Materials Project, which contains structural information of thousands of compounds [37]. Zhang et al. conducted a study using the Materials Project dataset to map potential DP compositions based on their stability and band gap (see Figure 6) [38]. The study found that as the atomic weight of A increased from Li to Cs, the thermodynamic stability also increased while decreasing X became heavier. As for M, the stability of the DP decreased in the column of alkali metals from Li to Cs, while M′ had less of an impact on stability [38]. The results of these Density Functional Theory (DFT) calculations and data from a plethora of sources were employed in developing machine learning models for predicting thermodynamical stability for lead-free halide perovskites; its general workflow is depicted in Figure 6 [39,40,41].

### 2.2. Electronic and Optical Characteristics and Application of Halide DPs

Numerous elpasolites have been synthesized and studied, and they are reported to have large bandgaps; their magnitude is usually ranging from 2 to 3.4 eV [5]. The nature of the bandgap for halide DPs may be tunable since both M and M′ contribute to the valence band maximum (VBM) and conduction band minimum (CBM) independently [33]. One way to classify these materials is to consider the valence electrons of ions M and M′. The bandgap is direct if both M and M′ have lone-pair electrons, or if both do not have lone-pairs and for vacancy-ordered DPs (Type I and Type III in Table 2). If only M or M′ have lone-pair electrons such as Cs_2_AgBiBr_6_, the bandgap is indirect (Type II in Table 2). In this case, the bonding orbitals of M(*n*d), M′(*n*s), and X(*n*p) contribute to the VBM, while the CBM minimum is dominated by the antibonding orbitals M′(*n*p) and X(*n*p) [42]. Table 2 shows these groups with examples from experimental publications and proposed application areas [19].

Differently from the standard AMX_3_ perovskites, the lattice tilts and octahedral distortion caused by the altering of ion A have no significant effect on the bandgap in A_2_AgBiBr_6_ [43]. However, in general, the effect of the substitution of A in DPs is less studied. Since the X ions strongly influence the VBM, it would be one simple method to tune the band structure via replacing or intermixing different halogen ions in the crystal structure, but this strategy fails in a lot of cases due to stability issues, and the most studied elpasolite structures are chloride-based, and very few are bromide-based [33]. It has been demonstrated that a stable DP structure can be built with iodine in the case of Cs_2_NaBiI_6_, and this solid shows a bandgap of 1.66 eV [44]. One extensively studied 2D perovskite-related system with iodine octahedra building blocks is Cu_2_AgBiI_6_, which is proposed to be a candidate for indoor photovoltaic absorbers [45,46].

**Figure 7:**
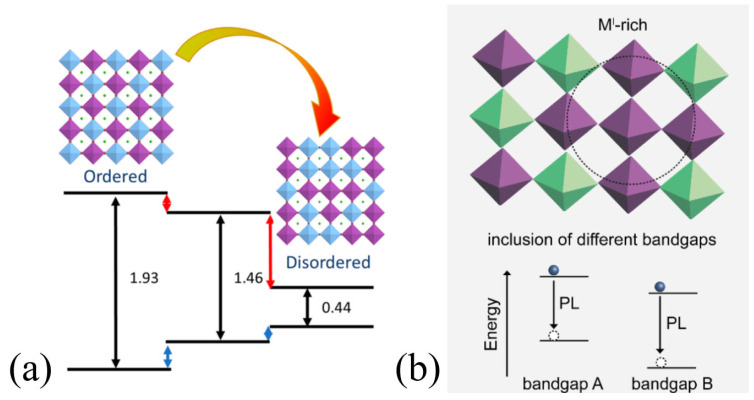
(**a**) Band alignment of the ordered, partially disordered, and fully disordered Cs_2_AgBiBr_6_ [47]. Copyright © 2017 American Chemical Society. (**b**) Formation of local domains leading to multiple emissive domains in Cs_2_AgBiBr_6_ [42]. Copyright © 2022 the authors. Published by American Chemical Society. This publication is licensed under CC-BY 4.0.

In addition to the comprising elements, the band structure could be significantly changed by the control of atomic arrangement via ordering parameters at the M-M′ sublattices, as illustrated in Figure 7 [47]. However, in most cases of DP compositions, the rock-salt order is thermodynamically favored due to the large charge difference between M and M′ and Pauling’s fourth rule. In rock-salt-ordered DPs, the high-valency +3 charged cations inside the octahedral halide cage are the furthest apart from each other, and due to energy or symmetry mismatch between the neighboring M(I) and M(III) frontier orbitals, this leads to (quasi)-0D electronic dimensionality. Therefore, the band edges are relatively localized [30]. Among these perovskites, the unique temperature-dependent light absorption and photoluminescent properties of published double perovskites are currently explained with different mechanisms; one of them is related to the question of atomic arrangement. The observed tendency is that lowering the temperature causes a blue shift of absorption onset and a red shift of emitted light. One of the proposed mechanisms behind this phenomenon is attributed to a local disordered phase, which could establish sub-bandgap emissive states (see Figure 7) [42,48].

One example of a non-rock-salt-ordered structure is Cs_2_Ag(I)Pd(II)Br_5_, which shows an indirect bandgap of 1.33 eV. Still, because of altered stoichiometry, strictly speaking, it does not belong to the group of perovskites [30]. On the other hand, if M′ is changed to a vacancy (see Figure 3 for the names of the branches of perovskites), resulting in low electronic dimensionality, some representatives may still exhibit dispersive bands and low bandgaps [5]. For instance, Cs_2_SnI_6_ has a relatively low bandgap and was proposed to be applied in thin-film transistors [49]. Its stable mixed-halide derivative, Cs_2_SnI_3_Br_3_, has been introduced in a dye-sensitized solar cell as a hole-transporting material, and the device reached 3.63% PCE [50].

**Figure 8:**
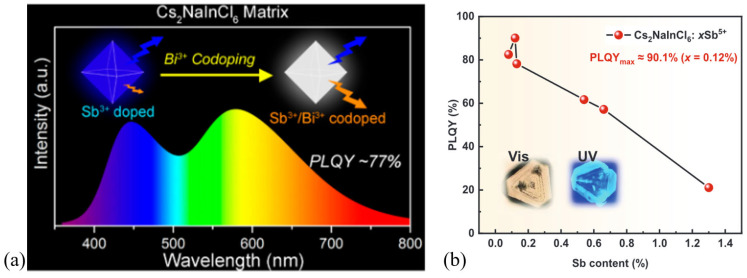
(**a**) White light PL in Sb^3+^/Bi^3+^ co-doped Cs_2_NaInCl_6_ microcrystals [51]. Copyright © 2021 American Chemical Society. (**b**) PLQY of pentavalent-doped Cs_2_NaInCl_6_ SCs [52] © 2023 Wiley-VCH GmbH.

Type III DP (Table 2) crystals have such symmetry conditions that certain optical transitions become symmetry-forbidden (inversion-symmetry-induced parity-forbidden) between CB and VB edges [19,43]. However, several compositions were published as host lattices from this group incorporating homo- and/or heterovalent dopants, which effectively modify the local crystal symmetry and crystal field intensity and result in radiative transition. For example, Figure 8a shows the dual-emission spectrum of Sb^3+^ and Bi^3+^ co-doped Cs_2_NaInCl_6_ microcrystals by Zhou et al. [51], and Figure 8b shows the photoluminescence quantum yield (PLQY) of blue emission by Sb^5+^-doped Cs_2_NaInCl_6_ single crystals (SC) by Liu et al. [52]. From the aspect of PLQY, some of these systems show remarkable values, for instance, a system of Cs_2_KInCl_6_:5%Sb^3+^ had 93% PLQY [53].

From an application standpoint, DPs have great potential in down-conversion LEDs; Table 3 summarizes numerous device structures featuring powder-based DP compositions for white light emission. In addition to WLEDs, certain DP compositions may be applied as NIR emitters, as was demonstrated by chromium-doped Cs_2_AgInCl_6_ crystals [54]. The current challenge of these microcrystalline DPs is to improve operational stability and to achieve a better understanding of charge injection, transport, and recombination [27].

To the best of our knowledge from a research perspective, spectroscopic studies of these systems date back to the 1970s, and until today, several questions have been raised and several energy transfer and quenching mechanisms have been proposed [51,53,56,64,65,66,67,68]. Building on one of these models, a structure–property descriptor was developed for one dopant, Sb^3+^ for machine learning algorithms, aiming at halide DP design [69]. Metal ion doping/alloying of the DP lattice, however, in addition to bandgap tuning and light-emissive behavior, may lead to other interesting properties, for instance, magnetic response [21]. However, the local environment of dopants/alloying elements might be different from the targeted [70,71]; thus, correct charge states and crystallographic positions of substituted metal ions need to be systematically investigated for halide DPs [72].

In DP nanocrystals (NCs), weak quantum confinement effects can be achieved if the Bohr radius is around 1 nm (e.g., 1.02 nm and 0.82 nm for Cs_2_AgSbCl_6_ and Cs_2_AgInCl_6_, respectively), giving similar electronic characteristics as for the 10 nm NCs compared to bulk materials [73,74]. Just like DPs in other forms (e.g., polycrystalline films), DP NCs have been universally applied for bright white emissions, solar cells/concentrators, photocatalysis, and X-ray imaging [74,75,76,77,78,79]. In addition, DP NCs share the basic properties of all NCs, like the advantage of being applicable for large-area white light emissions. The performances of recently developed DP NC-based devices in the abovementioned application areas are summarized in Table 4.

To summarize, so far, from the point of application, only 7% of reported lead-free perovskites were double perovskites used for solar cells (the PCE of a champion device with one of the most studied DPs, Cs_2_AgBiBr_6_, is 3.31%) [95]. Still, beyond photovoltaics, the potential is significantly larger. A total of 51% of reported lead-free halide DPs were used for developing LEDs, and 45% were used for photocatalysts [5,96]. A couple of representatives have also been proposed for NIR [70] and X-ray [19,97,98] detection. In the latter application area, an excellent review article was published in 2023 comparing the performances of different candidates for ionizing radiation detection [99]. In addition to these fields, certain DP systems were proposed to be employed in memristors [100], and spintronics [21,72], and be exploited for their thermochromic [101,102] behavior in thermometers, temperature sensors, or smart windows.

### 2.3. Manifesto of DPs: Synthetic Techniques and Technological Challenges

**Figure 9:**
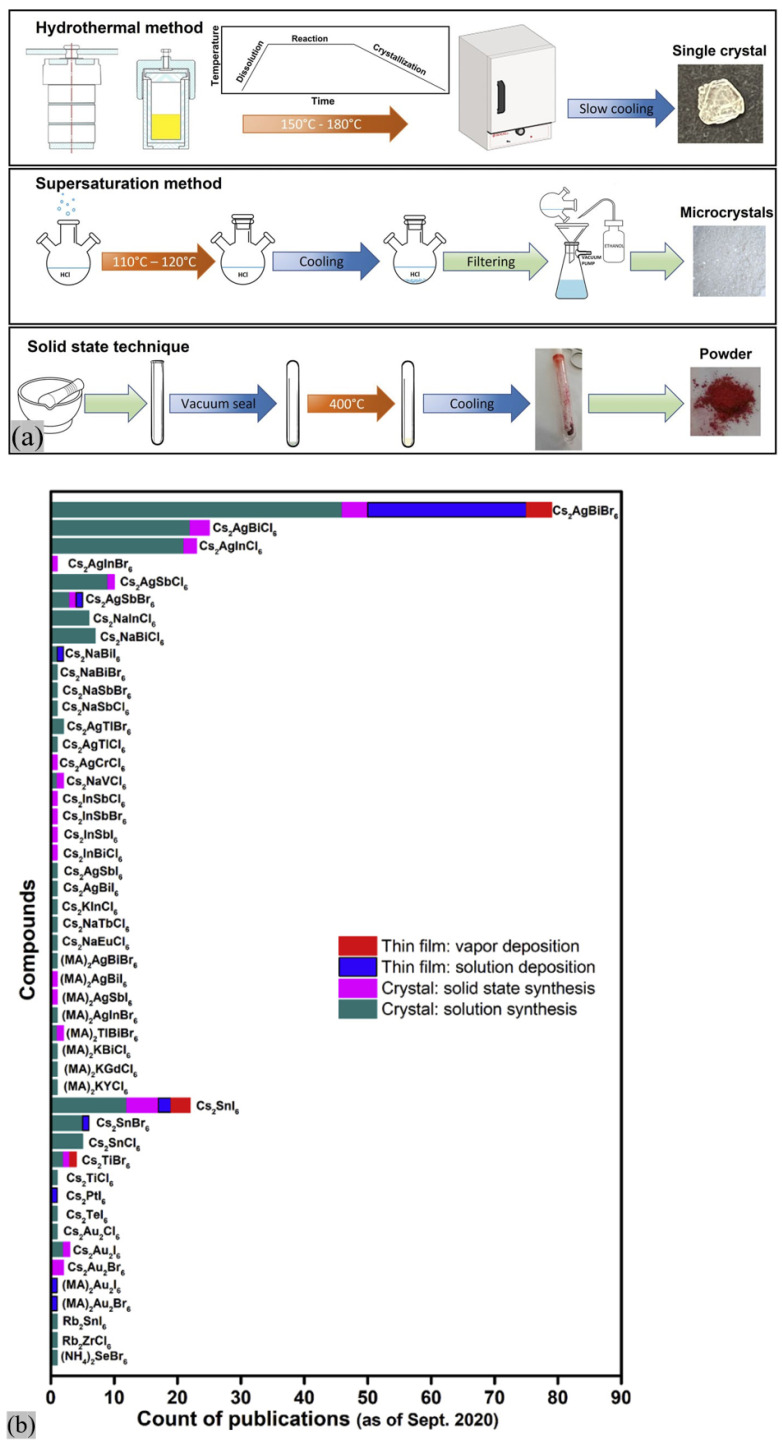
(**a**) Different approaches for the synthesis of DPs [63]. Copyright © 2023, AIP Publishing. This publication is licensed under CC-BY. (**b**) Publication statistics are achieved by text mining for different synthetic routes of numerous halide DPs [15]. Copyright © 2021 Elsevier Inc.

One of the advantageous properties of DPs is good stability under ambient conditions, and they may be synthesized in several forms. Figure 9 shows the three most used methods for the synthesis of these complex compositions: hydrothermal, supersaturation, and the solvent-free solid-state method [96]. Less common ways, but also solvent-free methods, include mechanochemical synthesis [103,104], as well as the melt-based Bridgman technique [105]. The hydrothermal crystal growth of DPs occurs in an aqueous mixture of inorganic salts in a sealed autoclave reactor, for which the system heats up to the reaction temperature and then slowly cools down. In the case of the supersaturation technique, the precursor is prepared at ambient conditions and heated up to a generally lower temperature than in the previous technique. The heat-up is subsequently followed by a cool-down step, which results in microcrystals [96]. The solid-state reaction mixture is sealed in a fused silica ampule under low pressure and heated up to high temperatures without solvents [106]. The Bridgman technique also occurs in a sealed silica tube filled with the precursor, but the tube is moved along the temperature gradient [107]. The mechanosynthesis employs a high-energy ball mill to obtain polycrystalline powders, and except for the intrinsic heat generated by the ball milling process itself, the DPs may be formed at room temperature [103]. Figure 9b shows publication statics on different synthetic routes for several DPs [15].

For the growth of SC DPs, either the hydrothermal reaction, solventless molten-salt medium synthesis, or the Bridgman method is applied. Due to random nucleation and isotropous growth, the hydrothermal method is difficult to scale up for large-scale thin-film production, but via surface engineering, remarkable results have been achieved and published [108]. The method depends on the solubility of inorganic salts in water at different temperatures and vapor pressures, and the formation is complex and relatively slow [109]. However, this facile method can also be applied for the synthesis of complicated composite structures like the DP Cs_2_AgBiBr_6_ supported on nitrogen-doped carbon materials for hydrogen evolution [110]. The family of solid-state techniques includes the so-called vapor transport method, which results in high-quality SCs, but it is a less-explored technique [99]. The Bridgman technique was introduced for the fabrication of high-quality perovskite ingots on a large scale [111]. The drawbacks of this technology are high energy demand, mechanical stress, high density of grain boundaries at the surface in close contact with the silica tube, and the breakage of SCs due to subsequent mechanical cutting [99]. For further readings, recently a comprehensive review has been published discussing different SC growth theories and techniques and showing countless examples from lead halide perovskites [107].

Another huge challenge of the development of lead-free halide DP-based devices is that they can be synthesized from the acid solution, but this is not suitable for thin-film device fabrication. In comparison to lead perovskites, they have higher formation temperatures and low solubility, and halide DPs show a quick crystallization rate, which complicates the uniform film formation. This may be circumvented by thermal evaporation, antisolvent engineering techniques, and post-annealing [33,43,112]. Moreover, different from the formation of films or SCs, DP NCs are usually synthesized with the assistance of ligands. Thanks to ligands, which reduce surface energy, doped DP nanocrystals obtain enhanced stability compared to polycrystalline films or SCs [113,114].

**Figure 10:**
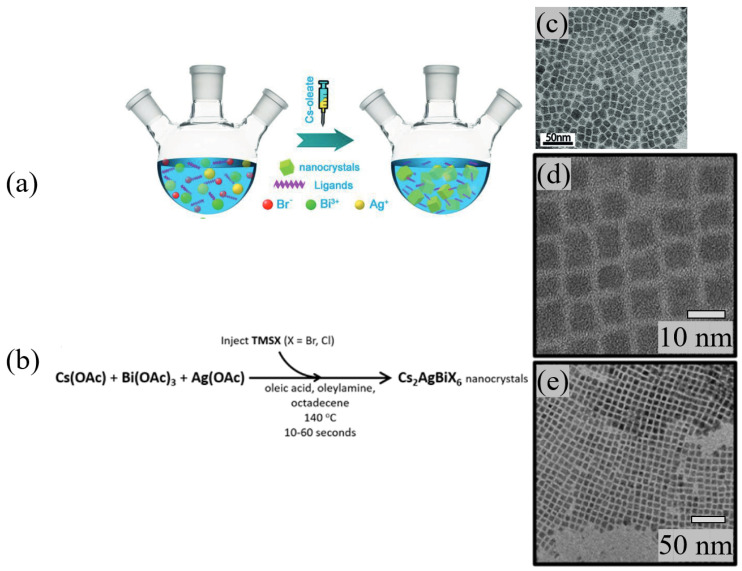
Two examples for the synthesis of NCs: (**a**) schematic illustration of the solution phase synthesis of Cs_2_AgBiBr_6_ NCs (hot injection route) [114]. © 2018 WILEY-VCH Verlag GmbH & Co. KGaA, Weinheim. (**b**) Synthesis of Cs_2_AgBiX_6_ NCs by Creutz et al. [115]. Copyright © 2018, American Chemical Society. (**c**) TEM image of Cs_2_AgBiBr_6_ NCs prepared with method (**a**). (**d**,**e**) TEM images of Cs_2_AgBiBr_6_ NCs prepared with method (**b**).

There are mainly two synthesis methods for DP nanocrystals: the hot injection (HT) method and the ligand-assisted reprecipitation (LARP) method. Synthesizing Cs_2_AgBiBr_6_ and Cs_2_AgBiCl_6_ by HT was first reported in 2018 by two different groups, and their approaches are depicted in Figure 10 [114,115]. Sidney E. Creutz et al. injected trimethylsilyl halide (TMSX) into octadecene (ODE) with the precursor metal acetates and ligands (oleic acid (OA) and oleylamine (OAm)) at 140 °C and achieved well-defined cubic Cs_2_AgBiBr_6_ and Cs_2_AgBiCl_6_ NCs with sizes of around 8–9 nm [115]. Lei Zhou et al. injected Cs-oleate into a high-boiling-point solution of BiBr_3_ and AgNO_3_ at 200 °C and achieved Cs_2_AgBiBr_6_ NCs with a size of 9.5 nm in cubic shape [114]. According to the report of Bekenstein et al., the two methods yield similar nanocrystals [116]. All in all, HT has been broadly applied for synthesizing DP NCs since 2018.

LARP was first reported by Yang et al. for the synthesis of DP nanocrystals with the following procedure: all the precursor materials were dissolved in a good solvent and injected into an antisolvent to form DP NCs, which turned out to be less regular than that achieved by the HT method [81]. They also found that the addition of OA would enhance the photoluminescence of DP NCs by 100 times through defect passivation, and afterward, OA is used in the LARP synthesis by default [117].

In the past five years, a lot of efforts have been made to dope/alloy metal ions in DP NCs to tune their optical properties [55,77,80,81,82,86,118,119]. Among these, indirect to direct bandgap tuning is achieved by In^3+^ doping in the case of Cs_2_AgIn_x_Bi_1-x_Cl_6_ NCs (x = 0.75 and 0.9) [81]. In addition, Han’s group has greatly improved the photoluminescent properties of DP nanocrystals by doping: after doping with Sb^2+^, green emission with unity PLQY is achieved for Cs_2_KInCl_6_ DP NCs, and further Mn^2+^ doping leads to white light emission with PLQY of 87% due to efficient energy transfer [77].

Besides the abovementioned doping/alloying method, ligand engineering in Cs_2_NaInCl_6_/Cs_2_AgInCl_6_ synthesis, e.g., by trioctylphosphine (TOP), has also been studied to improve photoluminescence and colloidal stability [83,87,120]. Furthermore, an interesting study on morphology control in DP NCs was also developed by Liu et al. Cs_2_AgBiX_6_ (X = Cl, Br, I) two-dimensional nanoplatelets were first reported, which showed better catalytic performance for CO_2_ photoreduction than their nanocube counterpart [74].

The synthesis of nanomaterials with quaternary elements is complicated and requires careful compositional tuning to ensure product purity. Despite this, a lot of achievements have been reached in DP NCs during the last five years. In the future, the synthesis method is expected to be broadened, e.g., the passivation of halide defects at the surface [121], and morphology engineering, to improve the stability for various applications of DP NCs.

## 3. Low-Dimensional Lead-Free Halide Perovskite Derivatives

Not featuring the corner-sharing network of octahedra, strictly speaking, the following classes of materials do not belong to perovskites. However, some representatives have similar crystallographic building blocks that otherwise cannot form stable perovskite structures, and some are derived from perovskite structures; thus, one can say that research on them is closely related to the perovskite field. Here, we include recent advances in the field of lead-free hybrid organic–inorganic 2D (fully inorganic 2D structures are out of the scope of this work [23]) and 0D perovskite-related material.

### 3.1. Hybrid Organic–Inorganic 2D Layered Halide-Perovskite-Related Structures

Hybrid organic–inorganic layered perovskites have been explored since the 1990s and are a promising class of material with potential applications as a surface passivation layer in 3D metal lead-based and lead-free halide photoabsorbers [122,123,124], thermoelectric energy conversion [125], photodetectors [126], magnets [127], memories [128,129], spintronics [130], energy storage, and perhaps many more [131]. Their versatility lies in their rich compositional diversity and structural tunability.

**Figure 11:**
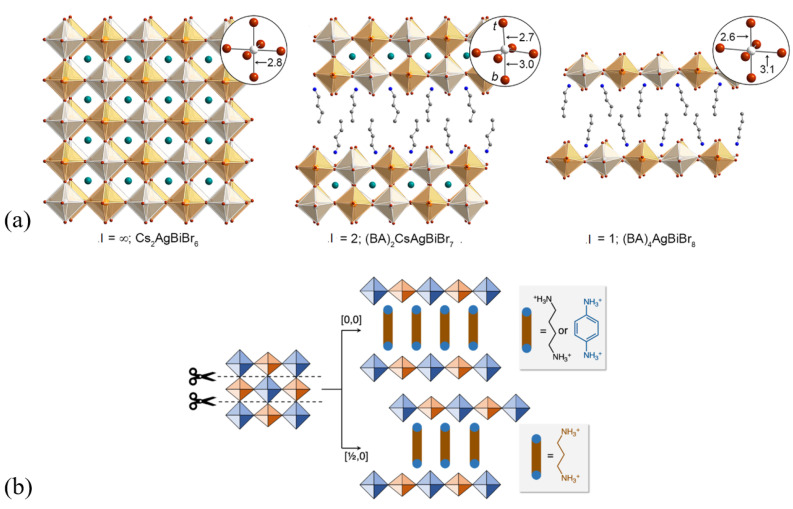
(**a**) Single-crystal structures at 298 K of the (001) layered double perovskites (BA = CH_3_(CH_2_)_3_NH_3_^+^). Insets show the Ag^+^ coordination sphere with selected bond distances in Å, t denotes terminal bromide, and b denotes bridging bromide [132]. Copyright © 2018, American Chemical Society. (**b**) [0,0] and [1/2,0] stacking patterns in DJ-layered DP derivatives [136]. Copyright © 2022, American Chemical Society.

If ion A in the perovskite composition is fully or partially changed to an organic cation that has one or two ionically interacting terminal functional groups (most frequently amines) and is larger than 2.6 Å, the 3D crystal structure may reduce along the (001) plane into slabs of 2D perovskite layers separated by organic layers (there are examples of (110) and (111) oriented structures; they are not discussed here) [23,132,133]. The organic spacers containing hydrophobic alkyl chains ensure protection against moisture and oxygen, thus being more stable than their 3D counterparts, but anisotropic 2D crystals are challenging to synthesize in the form of thin films [131,134,135]. These structures can be described with (S)_x_A_n−1_M_n_X_3n+1_ stoichiometry, where S is the templating organic cation spacer (if monofunctional, x = 2, if bifunctional, x = 1). If not fully replaced by S, A is the cation incorporated into the octahedral cavities [131,132]. Figure 11a aids in visualizing the dimensional reduction and consequent perovskite lattice distortion through the example of the incorporation of butylammonium (BA) ions in lead-free halide DP Cs_2_AgBiBr_6_ [132]. The thickness of the inorganic sheet (number of {MX_6_} slabs) may be one grouping factor since it determines the phase stability and optoelectronic properties greatly, for instance, the indirect–direct character of bandgaps may be modified by dimensional confinement [131,132].

Figure 11b shows another way of classifying these compounds according to the relative crystallographic phases of the inorganic perovskite layers: in the case of Ruddlesden-Popper (RP) perovskites, the adjacent perovskite layers are displaced by half a crystal unit cell along both in-plane directions (commonly involving monofunctional organic bilayers), while Dion–Jacobson (DJ) phases are defined by no in-plane displacements (mostly having bifunctional organic layers in their structure) [131]. In the case of DJ DP derivatives, due to the alternating cations, the stacking patterns can be formed in two ways, i.e., [0,0] or [1/2,0] [136]. From the aspect of dimensional tuning, there is a third group of structures called the alternating cation interlayer (ACI), where the organic interlayer space consists of different cations in an alternating pattern (guanidinium ion and methylammonium ions are reported to be such cation combinations that may form this specific structure), for which the structure leads to higher crystal symmetry and a narrower optical gap in comparison to the RP perovskite derivatives [131,133,137,138].

**Figure 12:**
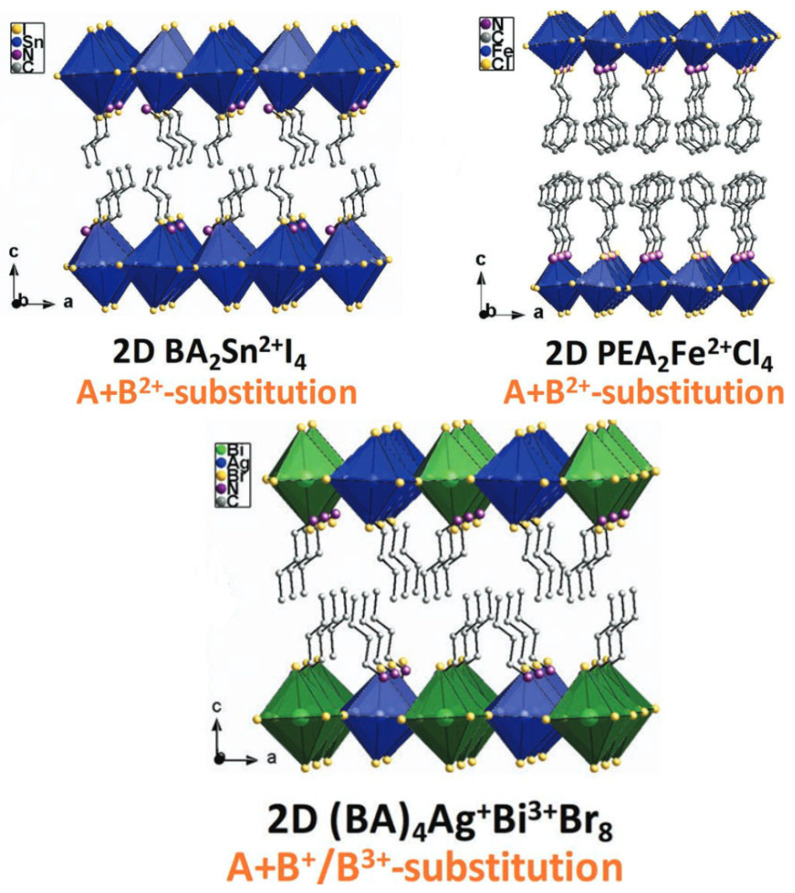
The different types of (001)-layered perovskite structures [23]. © 2019 WILEY-VCH Verlag GmbH & Co. KGaA, Weinheim.

Two-dimensional layered structures may allow for ionic compositions in inorganic slabs that otherwise do not form phase pure 3D crystals. One example is the DP combination of Ag^+^ and Sb^3+^ in RP (BA)_2_Ag_0.5_Sb_0.5_Br_4_ [135,139]. Molybdenum can be preserved in oxidation state +3 and combined with monovalent silver in (PPDA)_2_AgIMoIIICl_8_ [136] (PPDA = *para*-phenylenediammonium). Despite the rarity of silver in octahedral iodine coordination, (AE2T)_2_AgBiI_8_ [140] (AE2T = 5,5′-diylbis(aminoethyl)-[2,2″-bitiophene]) is synthesizable. A third way of classifying these crystals is to group them according to the constituting inorganic ions, which is shown in Figure 12: the DP derivative (S)_4_M^+^M′^3+^X_8_ and the (S)_2_M^2+^X_4_-type with Group IVA-type metals (Sn^2+^ and Ge^2+^) and with other divalent metal ions (such as Cu^2+^, Fe^2+^, Cr^2+^, etc.) [23].

**Figure 13:**
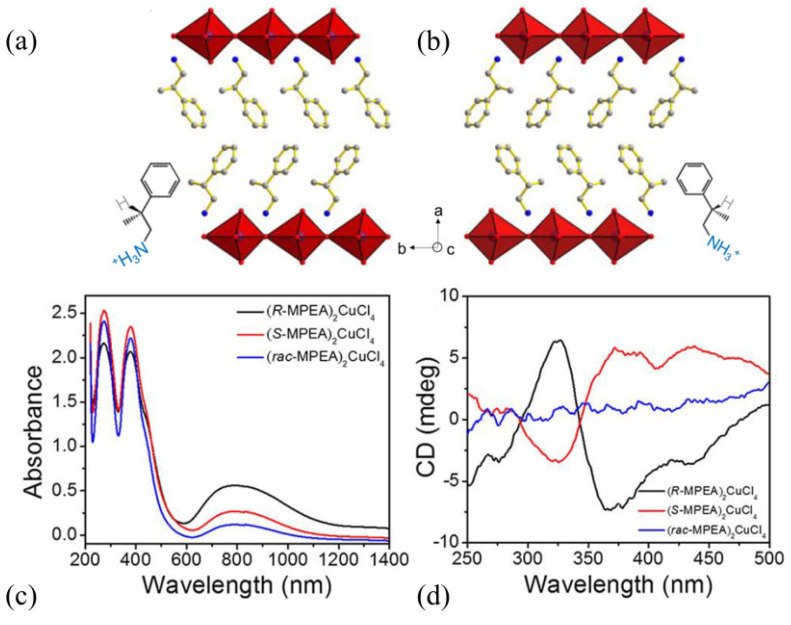
SC structures viewed along the c-axis (**a**) (R-MPEA)_2_CuCl_4_; (**b**) (S-MPEA)_2_CuCl_4_ [130]; (**c**) UV-vis-NIR absorbance spectra; and (**d**) circular dichroism spectra of abovementioned chiral perovskite derivatives [130]. Copyright © 2020, American Chemical Society.

By separating and connecting inorganic layers, organic spacers are important for determining the system’s functionality. Several different organic spacers have been introduced to these structures, which may even add to the functionality of the corresponding 3D analogs by being inherently electroactive, photoactive, chiral, or mechanochromic [131]. The incorporation of spacers that are insulating leads to natural quantum well behavior, where charges are confined to the inorganic layer [131]. Thus, one grouping point of these low-dimensional structures is the thickness of the inorganic sublattice with which the optoelectronic properties are gradually changing [132]. Using electroactive ligands such as functionalized thiophenes can modulate the electronic structure [131]. The superior optoelectronic characteristics of perovskites may be combined with chirality in these structures (3D perovskites likely remain in the theoretical development stage). If chiral spacers separate the perovskite layers, which leads to phenomena like circular dichroism, nonlinear optics or spin-related effects may be seen [141]. One example is shown in Figure 13, where enantiomeric 2D hybrid copper-based perovskites, (R-MPEA)_2_CuCl_4_ and (S-MPEA)_2_CuCl_4_, possess chirality confirmed by circular dichroism measurements (MPEA = β-methylphenethylamine) [130].

As proposed in this chapter’s introduction, these 2D structures have significant potential in wide application areas. From the materials design perspective, it is expected to be easier to engineer multifunctional structures based on low-dimensional structures employing A cations with functionalities [23]. Screening these compounds due to their complexity and diversity of their composition is challenging. Li et al. published a DFT-based hierarchical progressive screening method on 3D bulks to compare derived layered structures from the point of view of stabilities and electronic properties [142]. They found that (CH_2_)_8_(NH_3_)_2_Cs_n−1_Sn_n_Br_3n+1_ stoichiometry covers a class of material predicted to have high PCE, small carrier effective masses, and good stability and appropriate bandgaps [142]. To aid the discovery of new materials belonging to this group, Chen et al. screened 1,4-butanediamine-templated (BDA) DJ DP for solar application [143]. They published a map of decomposition enthalpies that were calculated considering the decomposition pathways from the desired compound into the corresponding common binary decomposition products. In general, they concluded that all combinations of (BDA)-templated halide structures Na^+^/K^+^/Rb^+^/Cu^+^/Ag^+^/Au^+^ and Bi^3+^/In^3+^/Sb^3+^ all are thermodynamically stable [143].

### 3.2. Electronically Zero-Dimensional and Perovskite-Inspired Crystal Structures

#### 3.2.1. 0D Anti-Perovskites

**Figure 14:**
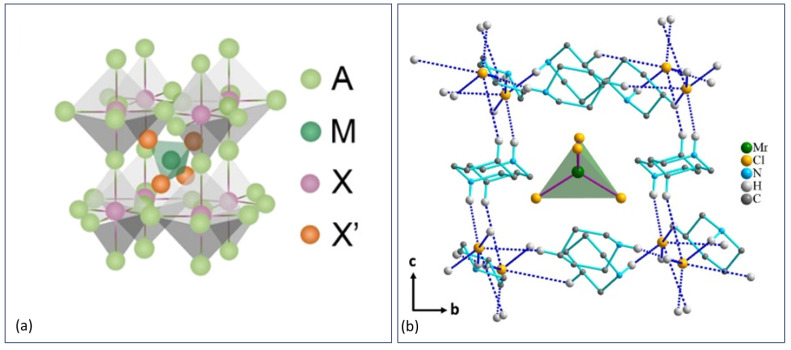
(**a**) An anti-perovskite structure with the formula [MnX’_4_]XA_3_, where [MnX’_4_] represents B. Reprinted (adapted) with permission from {ACS Energy Lett. 2021, 6, 5, 1901–1911}. Copyright {2023} American Chemical Society [146]. (**b**) Anti-perovskite with a 0D MnCl_4_ tetrahedra in the middle representing B. Reprinted (adapted) with permission from {Inorg. Chem. 2023, 62, 7, 3202–3211}. Copyright {2023} American Chemical Society [144].

Most 0D materials cannot be considered full members of the perovskite classification since the metal halide octahedral corner sharing is absent (see perovskite-inspired structures). However, 0D anti-perovskites do exist. The composition of anti-perovskites can be represented in several ways, one of them is X_3_BA, where X is a monovalent cation, A is a monovalent anion, and B is a divalent anion, or as [MX_x_]XA_3_, where M is a metal, X is a halogen, and A is a cation (see Figure 14a). Another way to represent anti-perovskites is A_3_BX, but this nomenclature is not so common in the optoelectronic field. The structure shows promising properties for optoelectronics [144,145]. Manganese (Mn)-based organic or inorganic metal halides are typical examples of when a 0D anti-perovskite structure can be favorable. Coupling between the Mn-Mn sites in an ordinary 3D perovskite quenches the emission, but in a 0D anti-perovskite, the distance between the Mn and Mn sites increases, resulting in limited coupling. Also, the confinement effect is enhanced by the structure and will improve the emission. Panda et al. synthesized a 0D anti-perovskite, (Piperidinium)_3_Cl[MnCl_4_] (see Figure 14b), with a PLQY of 54.5% [144], and Yan et al. synthesized six different Mn-cesium (Cs) halides, all with a PLQY over 80% [146] (see Figure 14a). The structure consists of octahedral complexes made up of a halide surrounded by organic monovalent cations, while the incorporated divalent anion consists of a tetrahedrally coordinated metal halide. This structure is represented as [MX_4_]XA_3_, where A is an alkali metal (I); M is a transition metal(II); and X represents Cl, Br, or I. In an anti-perovskite, XA_3_ builds up the classical corner sharing octahedra, while [MX_4_] represents the position in between the octahedra. However, not all [MX_4_]XA_3_ materials show an anti-perovskite structure. By comparing the structure of 22 different [MX_4_]XA_3_ materials, Yan et al. concluded that the ratio of half the tetrahedral edge length and the sum of the ionic radius of the A and X atoms had to be between 0.544 and 0.644 for 0D anti-perovskites [146].

#### 3.2.2. Perovskite-Inspired Structures

##### Definition of Different Crystals

**Figure 15:**
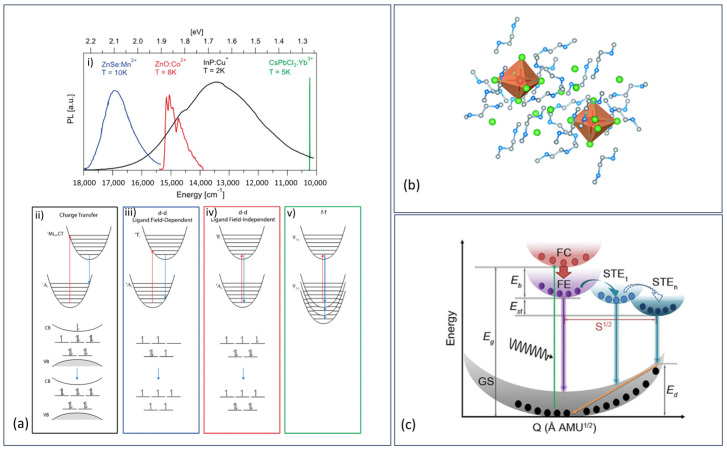
(**a**) (**i**) Gives an idea of the relative emission width of the different transition mechanisms seen in (**ii**–**v**). (**ii**) Metal–ligand charge transfer. (**iii**) d-d transitions with ligand field dependence and (**iv**) with ligand field independence. (**v**) f-f transitions. Reprinted (adapted) with permission from {Chem. Rev. 2023, 123, 12, 7890–7952}. Copyright {2023} American Chemical Society [165]. (**b**) SnBr_6_^4−^ octahedra surrounded by C_4_N_2_H_14_Br^+^ ligands, reproduced from Chem. Sci., 2018, 9, 586–593 with permission from the Royal Society of Chemistry [163]. (**c**) Schematic energy curves of STE formation. GS = ground state; E_g_ = the bandgap; E_b_ = exciton binding energy; E_st_ = self-trapping energy; FC = free carrier; FE = free excitons; S = Huang–Rhys factor; E_d_ = lattice distortion energy. Reproduced from [168]. © 2022 Wiley-VCH GmbH.

0D inorganic–organic hybrid metal halides, as well as fully inorganic metal halides (called 0D metal halides in the rest of the text), consist of the same type of building blocks as 3D metal halide perovskites used in optoelectronic devices such as solar cells and LEDs. However, for the structure to be considered electronically 0D, the octahedra in the perovskite must be separated to limit the metal–metal coupling. Separated octahedra means no corner sharing, and in this way, the perovskite structure is broken and should theoretically not be called perovskites (see Figure 15b). Only increasing the size of the A site molecule/atom in a 3D perovskite until the octahedra is separated results in the composition AMX_6_, where A is a large monovalent cation, M is a 5+ metal such as Bi^5+^, Sb^5+^, or V^5+^, and X is a halogen [147]. The octahedra can also be separated by 2, 3, or 4 A site monovalent molecules/atoms per unit cell, resulting in the compositions A_2_MX_6_ (M = +4) [148], A_3_MX_6_ (M = +3) [149], and A_4_MX_6_ (M = +2) [150].

So far, we have only discussed metal halides containing octahedral polyhedrons, but also materials containing tetrahedral or square pyramidal [151], or a mixture of tetrahedral and trigonal bipyramidal–polyhedral, can be perovskite-inspired materials. For example, tetrahedral-containing or seesaw-shaped materials with a divalent metal M result in the composition A_2_MX_4_ (M = 2+) [152]. Additionally, the A site molecule does not necessarily have to be monovalent, once again changing the A-M-X composition.

As a general conclusion, 0D metal halides consist of anionic metal halide polyhedrons spaced by large cations [153]. The general composition can be described as A_i_M_j_X_k_, a variety of combinations are included, and many of them have been called 0D perovskites, although the structures deviate a lot from the perovskite structure.

Another quite common feature in 0D metal halides is the incorporation of water molecules, sometimes becoming a part of the polyhedron, which then affects the coordination chemistry [154]. By divagating slightly from the 0D structure, we will find structures where two or more metal halide polyhedrons will cluster up, showing corner, edge, or face sharing. This structure can sometimes be called quasi-zero-dimensional (q-0D) structures. The terminology around q-0D is not fully established, and sometimes the quasi term can be used to point out that the type of 0D materials that have been discussed in this section are not 0D in the same sense as quantum dots since they can form macroscopically sized SCs. The 0D structure for this for the materials discussed here is only 0D in electronic matter [155].

##### Properties

As seen in the sections above, there are many kinds of 0D metal halides, and all of them will not have the same properties. However, they will be more stable than their 3D counterparts since the large organic (or inorganic) cations will act as a protective layer surrounding the metal halide polyhedrons. Electronically low-dimensional materials often have hampered charge transfer and independent luminescent centers that are relatively insensitive to long-distance chemical morphology, resulting in rather stable luminescence. They also have a stronger quantum confinement effect, resulting in closely localized excitons with high exciton binding energy, leading to high PLQY [156,157].

The light emission in 0D metal halides can originate from self-trapped excitons (STE), d-d transitions, d-f transitions, metal–ligand charge transfers (MLCT), defect states, as well as mixtures of these and other emission routes [158,159]. STEs are formed by local lattice distortion induced by the excited state, generating a distortion field trapping the exciton that induced the distortion in the first place (self-trapped). The photoluminescent emission energy can be expressed as E_PL_ = E_g_ − E_b_ − E_st_ − E_d_, where E_g_ is the bandgap energy, E_b_ is the exciton binding energy, E_st_ is the self-trapping energy, and E_d_ is the lattice deformation energy. Jahn–Teller distortion is the most common cause of self-trapping in perovskite/perovskite-like structures, while the formation of V_k_ centers is the most common cause in alkali halides. The formation of STEs is increased in low-dimensional crystal structures, making it a common origin for emission in 0D metal halides. Emission by STE gives a broad PL spectrum and large stokes shifts (example: (C_4_N_2_H_14_X)_4_SnX_6_) (see Figure 15b,c) [160,161,162,163]. d-d transitions can mainly be seen from transition metals surrounded by ligands, and emission energy originates from the crystal field splitting of the otherwise degenerated d orbitals. d-d transitions with ligand field dependency (changes in orbital occupancy) generate relatively broad emissions due to vibronic broadening, while transitions that are close to being independent of the ligand field (near unchanged electron distribution) reveal a much narrower emission (see Figure 15a) [164,165,166]. The emission becomes stronger when dipole–dipole coupling can be avoided between the metal centers, and therefore the strongest d-d emissions can be found within electronically 0D materials or from doped materials [167]. One of the most typical examples of d-d transitions in 0D metal halides is seen in manganese-based materials, where the octahedral polyhedrons emit weaker than the tetrahedral since the transitions are Laporte-forbidden (d-d and centrosymmetric), as well as oppose the spin selection rule. In contrast, the tetrahedral polyhedrons only oppose the spin selection rule since the shape is not centrosymmetric; nevertheless, both have forbidden transitions, which causes long lifetimes.

The d-f transitions are much faster than the d-d and f-f transitions since they are parity-allowed (example: Cs_3_CeI_6_) [169]. Charge transfer relaxation gives broad emission due to geometric distortions, as seen for the metal-to-ligand charge transfers (MLCTs) in Figure 15a(i,ii). MLCTs are not common in 0D metal halides, but there are some materials with ligand-to-metal charge transfer (LMCT)-based emission (example: (PTMA)_3_Cu_3_I_6_) [170] and MLCT-assisted emission (example: [(C_6_H_5_)_4_P]_2_SbCl_5_) [171].

As mentioned in the beginning of this section, the emission could also be from defect states. As an example, Ran et al. showed that the emission from (C_6_H_8_N)_6_InBr_9_ SCs originates from Br vacancies; however, owing to a strong thermal quenching effect, the material showed a low PLQY of only 2.7%. Yet when doping the material with antimony, creating (SbBr_6_)^3−^ octahedra, the PLQY increased to 71.8%. The increased PLQY originates from STE recombination localized on the (SbBr_6_)^3−^ octahedra and is enhanced by the charge transfer from the defects [172].

##### Applications

As for the 3D perovskites, 0D metal halides can be employed as the active layer in LEDs, but they are generally not suitable for solar cells due to high exciton binding energies and electronic confinements. There are several 0D material systems synthesized for LED applications, but one of the major difficulties with 0D-based electrically driven LEDs is low conductivity due to bulky spacing molecules, but with good material engineering and doping, the conductivity can be increased [173]. 0D materials also usually have large Stoke shifts, which is preferable for LEDs since this reduces reabsorption (see Table 5) [174].

Another more common application is to use them as phosphor materials in LEDs, i.e., the phosphor covers the lightbulb or diode surface of a high-energy emissive (UV or blue) diode that can excite the phosphor, which emits a lower energy wavelength. By mixing phosphors with different emission wavelengths, a white light emission can be achieved. 0D materials are often more stable than their 3D counterpart, while showing a high PLQY value, making them suitable as phosphorus materials (see Table 6). Phosphor materials can also be used in applications such as encoding and anti-counterfeiting or re-printable paper. Properties such as quenched emission in the presence of water or some other solvent, as well as dual or triple emission colors that emit stronger/weaker depending on the excitation wavelength, can be utilized for these applications [161,182,183]. X/β-ray scintillators made from 0D metal halides are an emerging field with promising results [184]. There are also reports of fluorescent sensors used for detecting heat, moisture, or specific solvents (see Table 6) [185,186].

##### Synthesis

One common way to synthesize perovskites as well as perovskite-like materials is by different solution-based methods. The A site molecule/atom or A site halogenated salt is dissolved together with B site halogenated salt or oxide. Sometimes, HI, HBr or HCl is added for excess halogens to enhance the reaction. When the solvent has evaporated, cooled down from a higher temperature (see Section 2.3), or an antisolvent has been diffused into the solvent, the material will precipitate and crystallize [212].

0D metal halides can also be synthesized using mechanochemical techniques such as ball milling or grinding using a mortar; this will create a fine powder of the 0D material without using any solvents or by using only a small amount of solvent to enhance the reaction. The properties of the solution-grown materials and the mechanically grown materials can differ slightly [212].

An autoclave can be used to prepare the synthesis under thermally activated pressure. The chosen molar ratio of the A site and B site molecule/atom is added to the autoclave together with HI, HBr, or HCl depending on the preferred halogen. The B site precursor can be in the form of an oxide or halogenated. This technique can generate high-quality SCs [209,213]. Some materials are synthesized to become both nanocrystals and electronically 0D at the same time. For this type of material, hot injection synthesis is common (see Section 2.3).

**Figure 16:**
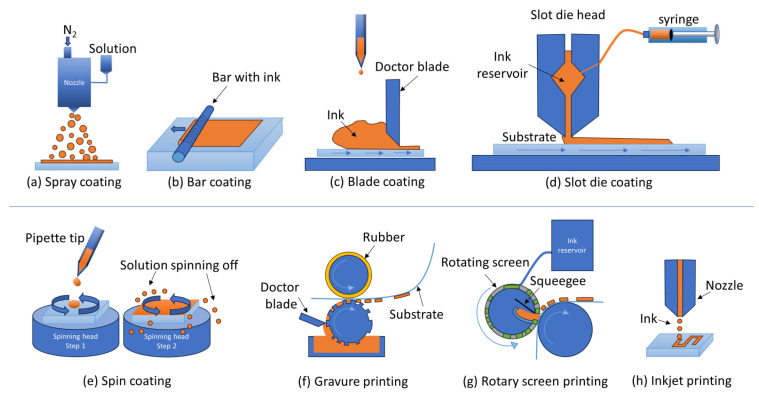
Schematic figures of most solution-based deposition methods used for perovskite. Note that the setup for the different techniques can vary. (**a**) Spray coating; (**b**) bar coating; (**c**) blade coating; (**d**) slot-die coating; (**e**) spin coating; (**f**) gravure printing; (**g**) rotary screen printing; (**h**) inkjet printing.

For thin-film fabrication, spin coating is the most utilized method in research laboratories. Nonetheless, other solution-based techniques such as spray, dip, slot-die, bar, or blade coating, as well as gravure, screen, or inkjet printing, are also used (see Figure 16) [214]. Additionally, evaporation methods such as physical vapor deposition (PVD) and chemical vapor deposition (CVD) are applicable [169]. Spin coating is an easy and low-cost option and a well-operated coating results in high-quality thin films. The two main problems with spin coating are scalability and material waste; only 2–5% of the solution stays on the substrates. Other solution-based methods are also relatively cheap and more suitable for up-scaling, but so far, the best-performing devices are made by spin coating. The film quality from spray coatings is dependent on the size of the droplets, and it is hard to make the films uniform and with a precise thickness. An advantage of spray coating is the possibility of coating substrates with complex shapes [215,216]. Dip coating gives high crystallinity, but also relatively many pinholes and high surface roughness [217].

If spin coating is the most common coating method on a lab scale, slot-die coatings incorporated with roll-to-roll (R2R) functions are the most common for the industrial manufacturing of perovskite solar cells as well as organic photovoltaics. Slot-die coatings are material-efficient and can easily be made on flexible substrates, plus with the roll-to-roll function, the deposition becomes easily adapted to automatization [218]. Blade coating and bar coating are similar techniques to slot-die coating but with less ink control. The most important factor to receive good-quality films, while using any of the three techniques, is to control the evaporation [219]. Inkjet printing works as a common household printer, and one of the advantages of inkjet printing is high-resolution printing without the usage of masks; another is that the nozzle is never in contact with the substrate [220]. One disadvantage is the formation of the coffee ring effect which originated from increased evaporation on the droplet edge due to curvature and induced flow within the droplet [221]. This effect generates uneven coatings but can be avoided by controlling the evaporation speed concerning the diffusion rate of the solute [222]. Gravure printing is expensive due to equipment, has limited resolution, and is time-consuming, but it has demonstrated itself to be a method to consider by generating PCE values above 19.10%. Most of the abovementioned solution-based techniques have received PCE values higher than 19%: blade coating at 21.09% (2021), slot-die coating at 20.80% (2021), spray coating at 19.40% (2020), and inject printing at 21.60% (2021) [223]. Bar coating and screen printing are lagging behind with PCEs of 17.53% (2021) [224] and 15.89% (2020), respectively [223]. All these techniques are also possible to adopt to R2R [225,226].

In PVD, the material is evaporated in a high-vacuum chamber where it then sublimates on the substrate. The evaporation could be performed from one single source containing the full material or from several sources that can be evaporated sequentially or simultaneously. In CVD, gaseous phases are introduced into the chamber and react on the substrate. The precursors can be evaporated to decomposition to later form the same material again by chemical reactions on the surface, or two or several gaseous precursors react to form a new material. CVD is more complicated than PVD since reactions must form, and many atoms/molecules are needed for a high probability of the reaction. PVD is more expensive to operate than solution-based methods, especially due to the high vacuum needed for good depositions. Advantages are an even film thickness, good film quality, good thickness control, and the avoidance of harmful solvents [227]. Without trapped solvents, one source for generating defects is eliminated. Additionally, there are no solvents to dissolve previous layers.

Depending on the application, as well as the material, different deposition techniques can be preferred. While pinholes are very bad for optoelectronic devices, they do not matter as much for phosphorus materials. And, while the active layers in solar cells preferably would be thin, the layers for scintillators need to be much thicker. Some materials might be sensitive to heat, while others are sensitive to solvents. And sometimes a combination of coating techniques might be the best alternative.

## 4. Conclusions

Concerns have been raised regarding the toxicity and environmental impact of lead-based perovskite solar cells. Therefore, as a follow-up on explosive perovskite research, efforts have been made to replace lead while preserving the advantageous properties of this structure. The different approaches led to the expansion of the field to structures not strictly meeting the definition of perovskites but still showing some structural and characteristic similarities. In this review, we aim to classify the relatively less-researched examples of synthetic halide perovskites and related structures. In addition, we briefly introduce the mapping strategies, general optoelectronic properties, synthetic techniques, and possible application areas of uncharted classes, i.e., halide double perovskites, hybrid organic–inorganic layered 2D perovskite derivatives, and electronically zero-dimensional structures. Examples of recent advances in the classes presented indicate that more explorative research is needed to find new compositions, and we hope this viewpoint aids in the rational design of these compounds.

## Figures and Tables

**Table 1:** Generalized clinical symptoms of Pb poisoning in humans [13]. © 2020 by the authors. Licensee MDPI, Basel, Switzerland.

Body Organ/System	Clinical Symptoms of Pb Poisoning
Eyes	Blindness of parts of visual field Hallucinations
Ears	Hearing loss
Mouth	Unusual taste Slurred speech Blue line along the gum
Kidney	Structural damage and failure Changes in the excretory function
Liver	Jaundice Lead-induced oxidative stress Decreased liver function Microvesicular and macrovesicular steatosis Hemosiderosis and cholestasis
Skin	Pallor and/or lividity
Central nervous system	Insomnia Loss of appetite Decreased libido Irritability Cognitive deficits Memory loss Headache Personality changes Delirium Coma Encephalopathy
Reproductive organs	Sperm dysfunctions Pregnancy complications Preterm birth
Abdomen/Stomach	Pain Nausea Diarrhea Constipation
Blood	Anaemia
General	Malaise Fatigue Weight loss
Neuro-muscular	Tremor Pain Delayed reaction times Loss of coordination Convulsions Foot or ankle drop Seizers Weakness
Bones	Mineralizing bones and teeth Decreased bone density

**Table 2:** Double perovskites grouped into three classes according to the valence electrons M and M′ ions and their potential application areas [19] © 2018 Elsevier Inc.

A_2_M(I)M(III)X_6_	Optoelectronic Properties	Synthesized Compounds	(Potential) Application
Type I: s^2^ + s^2^	direct bandgap suitable bandgap values strong light absorption high electronic dimensionality expected defect tolerance	(MA)_2_TlBiBr_6_	(solar cell) (light-emission device)
Type II: s^0^ + s^2^	indirect bandgap large bandgap values reduced electronic dimensionality long carrier lifetime not good carrier transport	Cs_2_AgBiCl_6_ Cs_2_AgBiBr_6_ (MA)_2_AgBiBr_6_ (MA)_2_AgbiI_6_ Cs_2_AgSbCl_6_ (MA)_2_AgSbCl_6_ (MA)_2_KBiCl_6_ Cs_2_NaBiCl_6_	solar cell X-ray detector photocatalysis (X-ray imaging)
Type III: s^0^ + s^0^	direct bandgap dipole-forbidden transition large bandgap values reduced electronic dimensionality	Cs_2_AgInCl_6_ (MA)_2_KGdCl_6_ (MA)_2_KYCl_6_ Cs_2_NaGaF_6_	photodetector laser light-emission device
Vacancy-ordered	direct bandgap strong light absorption existence of deep mid-gap defects not good carrier transport	Cs_2_SnI_6_ Cs_2_PdBr_6_ Cs_2_Ti(Br/I)_6_ Cs_2_TeI_6_	solar cell light-emission device (X-ray imaging)

**Table 3:** Performance summary of powder based WLEDs [27] © 2023 the authors. Advanced functional materials published by Wiley-VCH GmbH.

Device Structure	PLQY [%]	CIE (x, y)	CCT [K]	CRI	Ref.
UV LED/Cs_2_AgIn_0.7_Bi_0.3_Cl_6_ NCs/PMMA	4	(0.36, 0.35)	4443	91	[55]
UV LED/Cs_2_NaInCl_6_:2.5%Sb, 45%Tb, 3%Mn NCs	74	(0.41, 0.39)	3371	89.2	[56]
UV LED/Cs_2_(Na, Ag)InCl_6_:7.09%Ho^3+^	57.09	(0.39, 0.46)	N/A	75.4	[57]
UV LED/Cs_2_Na_0.4_Ag_0.6_In_0.995_Bi_0.005_Cl_6_:Mn^2+^	31.8	(0.3784, 0.4216)	4323.4	82.6	[58]
UV LED/Cs_2_AgIn_1−x_Bi_x_Cl_6_	39	(0.417, 0.391)	3119	85	[59]
UV LED/Cs_2_Ag_0.4_Na_0.6_InCl_6_:1%Bi, 1%/BaMgAl_10_O_17_:Eu^2+^	98.6	(0.4, 0.38)	4430	95.7	[60]
UV LED/Cs_2_Ag_0.4_Na_0.6_InCl_6_:Bi, Gd/BaMgAl_10_O_17_:Eu^2+^	87.57	(0.3464, 0.3224)	4818	93.9	[60]
UV LED/Cs_2_Ag_0.7_Na_0.3_InCl_6_:Bi	87.2	(0.38, 0.44)	4347	87.8	[61]
UV LED/Cs_2_AgScCl_6_:0.05Bi	60	(0.366, 0.367)	4100	96	[62]
UV LED/Cs_2_Na_0.4_Ag_0.6_InCl_6_:Bi	73.3	(0.461, 0.443)	2930–6957	84.8–97.1	[63]

**Table 4:** Recent advances in DP NC-based optoelectronic devices and photocatalysts. (1) EL: electroluminescence (2) EQE: external quantum efficiency; (3) OQE: optical quantum efficiency; (4) PCE: power conversion efficiency.

Luminescence			
Composition	PL Peak [nm]	PLQY (%)	Ref.
Cs_2_AgInCl_6_:Mn	620	16.4	[80]
Cs_2_AgBiCl_6_:In	570	36.6	[81]
Cs_2_AgInCl_6_:Bi	580	11.4	[82]
Cs_2_AgInCl_6_:Na/Bi	600	40	[83]
Cs_2_AgBiCl_6_:Al	630	17.2	[84]
Cs_2_NaInCl_6_:Ag	535	31.1	[85]
Cs_2_NaInCl_6_:Sb/Mn	455/622	24	[86]
Cs_2_NaInCl_6_:Sb	448	50	[87]
Cs_2_KInCl_6_:Sb	515	95	[77]
Cs_2_KInCl_6_:Sb/Mn	510/630	87	[77]
Cs_2_NaYCl_6_:Sb	461	51.8	[88]
Cs_2_NaTbCl_6_:Sb	Multiple peaks (green emission)	24	[79]
Cs_2_ZrCl_6_	446	60.37	[89]
Cs_2_HfCl_6_	628	40.71	[90]
MA_4_InCl_7_:Sb	620	84	[91]
Light-emitting diodes			
	EL (1) peak [nm]	Efficinecy	
Cs_2_AgInCl_6_:Bi	557	58 cd/m^2^ (luminance); 0.01% (EQE (2))	[92]
Cs_2_AgInCl_6_:Na/Bi/Tb	610	2793 cd/m^2^ (luminance); 0.76% (EQE (2))	[93]
Photocatalysis			
Composition	Reaction	Efficiency	Ref.
Cs_2_AgBiX_6_ (X = Cl, Br, I)	CO_2_ photoreduction	0.035% (EQE (2))	[74]
Cs_2_AgBiBr_6_	NO removal	97% (removal rate)	[76]
Luminescent solar concentrators			
Cs_2_AgInCl_6_:Na/Bi		21.2% (internal OQE (3))	[75]
Solar cells			
Cs_2_AgBiBr_6_		0.46% (PCE (4))	[78]
Scintillator			
Cs_2_NaTbCl_6_:Sb		140 nGyair/s (detection limit)	[79]
Photodetector			
Cs_4_Cd_0.75_Mn_0.25_Bi_2_Cl_12_		0.98 × 10^4^ A W^−1^ (responsivity); 3 × 10^6^% (EQE (2))	[94]

**Table 5:** Electronically driven LEDs based on electronically 0D materials.

Material	Metal	Device Structure	EQE (%)	Emission Wavelength [nm]	Luminescence [cd m^−2^]	PLQY (%)	Comment	Ref.
(ABI)_4_MnBr_6_	Mn	ITO/PEDOT:PSS/poly TPD/(ABI)_4_MnBr_6_/TPBi/LiF/Al	9.8	629	4700	80	PEO 1 wt% additive	[174]
[PPh_4_]_2_[MnBr_4_]	Mn	ITO/PEDOT:PSS/TCTA:26DCZPPY (1:2):(Ph_4_P)_2_[MnBr_4_]/BmPyPb LiF/Al	9.6	518	2339		Active layer mixed with hole transport	[175]
DBFDPO-MnBr_2_	Mn	ITO/MoO_3_/TAPC/TCTA (50 wt%) DBFDPO-MnBr_2_/TmPyPB/LiF/Al	10.5	552		81.4	Active layer mixed with hole transport	[176]
(MePPh_3_)_2_SbCl_5_	Sb	ITO/PEDOT:PSS/Poly-TP/TAPC:2,6-DCZPPY:(MePPh3)2SbCl5 (6:3:1)/TPBi/LiF/A	3.1	593	3500	99.4	Active layer mixed with hole and electron transport	[177]
TPPcarzSbBr_4_	Sb	ITO/PEDOT:PSS/PVK/TPPcarzSbBr_4_/ZnO/LiF/Al	5.12	653 nm	5957 cd m^−2^	93.8%		[178]
Cs_3_Cu_2_I_5_:CsCu_2_I_3_	Cu	ITO/PEDOT:PSS, /Cs_3_Cu_2_I_5_:CsCu_2_I_3_/TmPyPB, /LiF/Al	3.1	565	1570	30	Mixture of 0D and 1D	[179]
TEA_2_Cu_2_Br_4_	Cu	ITO/PEDOT:PSS/TEA_2_Cu_2_Br_4_/TPBi/LiF/Al	0.11	463	85	94.73	[Cu_2_Br_4_] units	[180]
Cs_3_CeBr_6_	Ce	(ITO)/ZnO/Al_2_O_3_/Cs_3_CeBr_6_/TCTA/TAPC/HAT-CN/Al	0.42	391/421		91		[181]

**Table 6:** A selection of electronic 0D materials for various applications. Abbreviations: SSL = solid-state lightning, * LTC = lowering temperature crystallization.

Formula	PLQY (%)	Form	Valency	Em λ [nm]	Isolated Polyhedral	FWHM/ Stokes Shift [nm]	Method/ Application/ Emission	Ref.
Cs_3_Cu_2_I_5_	58	film	I	440	[Cu_2_I_3_]^−^	81/155	Thermal evaporation/LED/STE	[187]
** Cs_3_Cu_2_I_5_**	98.71	SC	I	443–456	[Cu_2_I_5_]^3−^	99/135	Annealed together/ /STE	[188]
[(C_3_H_7_)_4_N]_2_Cu_2_I_4_	91.9	SC	I	483, 637	[CuI_2_]^−^		Solvent evaporation/ /STE	[189]
**(Gua)_3_Cu_2_I_5_**	96	SC	I	481	[Cu_2_I_5_]^3−^	125/156	Heated solution/WLED/STE	[190]
Cs_4_SnBr_6_ with SnF_2_	62.8	powder	II	540	[SnBr_6_]^4−^		Ball milling/WLED/STE	[191]
**(C_4_N_2_H_14_Br)_4_SnBr_6_**	95	SC	II	570	[SnBr_6_]^4−^	105/215	Antisolvent diffusion/phosphor/STE	[163]
(C_4_N_2_H1_4_I)_4_SnI_6_	75	SC	II	620	[SnBr_6_]^4−^	118/210	Antisolvent diffusion/phosphor/	[163]
**(Bmpip)_2_SnBr_4_**	75	SC		666	[SnBr_4_]^2−^	69/326	LTC/X-ray scintillator/	[192]
Cs_3_ZnBr_5_	7.89	NC	II	468	[ZnBr_4_]^2−^	76/193	Hot injection/SSL, display X-ray scintillator/STE	[193]
**(ABI)_2_ZnCl_4_**	24.28	SC	II	395	[ZnCl_4_]^2−^	92	Solvent evaporation/Anti-Counterfeiting, X-ray scintillator, WLED/	[194]
(PMA)_2_ZnCl_4_	37.2	crystal	II	413, 440	[ZnX_4_]^2−^		LTC */WLED	[195]
** Cs_3_MnBr_5_**	48 (1.29 with 2H_2_O)	NCs	II	520	[MnBr_4_]^2−^	43	Hot injection/anti-counterfeiting/d-d transition, and possible STE	[196]
(C_9_NH_20_)_2_MnBr_4_	81.08	Crystal	II	528	[MnBr_4_]^2−^	64	LTC/sensor/d–d transition	[185]
**(Ph_4_P)_2_MnBr_4_**	98	Crystal	II	512	[MnBr_4_]^2−^	48/52	Diffusion, ball milling/WLED/d–d transition	[197]
(1-mPQBr)_2_MnBr_4_	60.70	SC	II	520	[MnBr_4_]^2−^	43	Solvent evaporation/ /d–d transition	[198]
** (TBA)_2_MnBr_4_**	93.76	SC	II	512	[MnBr_4_]^2−^	38.7/	Solvent evaporation/X-ray scintillator/d–d transition	[199]
Cs_3_BiCl_6_		NCs	III	391	[BiCl_6_]^3−^	60/59	Hot injection/ /STE	[200]
**Cs_3_Bi_2_Br_9_** **with oleic acid**	4.5	NCs	III	460	[BiBr_6_]^3−^	45	antisolvent injection	[201]
(PMA)_3_BiBr_6_	<1%	Crystal	III	405/510	[BiBr_6_]^3−^	153/160	LTC/ /STEs	[202]
** Cs_3_InBr_6_**	22.3	NCs, lower PLQY as SC	III	450	[InBr_6_]^3−^	/75	Hot injection/ /singlet and triplet STE/lighting, displays	[203]
Cs_2_InBr_5_(H_2_O)	33	SC	III	695	[InBr_5_(H_2_O)]^2−^	/340	LTC/Water sensor/STE	[204]
**(DETA)InBr_6_**	1.40 (24.12 Sb^3+^doping)	SC	III	400, 500-700	[InBr_6_]^3−^	134/200	LTC/ /STE	[205]
K_3_SbCl_6_ Mn^2+^-doped	22.3	NCs	III	440	[SbCl_6_]^3−^	102/120	Hot injection/WLED/STE and defect	[206]
**(C_9_NH_20_)_2_SbCl_5_**	98	SC	II	590	[SbCl_5_]^2−^	119/210	Antisolvent diffusion/phosphor/	[163]
TEBA_2_SbCl_5_	98	SC or powder	III	590	[SbCl_5_]^2−^	140/250	antisolvent diffusion or injection/WLED/STE	[207]
**(PPN)_2_SbCl_5_**	98.1	SC	III	635	[SbCl_5_]^2−^	142/225	Antisolvent injection/X-ray scintillator	[208]
(PPh_3_H]_2_SbCl_5_	74.50	SC	III	653	[SbCl_5_]^2−^	/283	Autoclave/ /singlet, triplet emission	[209]
**(NII)_2_SbCl_5_**	88.9	SC	III	610	[SbCl_5_]^2−^	118/248	Antisolvent diffusion/SSL/STE	[210]
(Bmpip)_2_GeBr_4_	1	SC	II	670	[GeBr_4_]^2−^	/330	Antisolvent injection/SSL, X-ray scintillator/singlet, triplet exciton emission, lone pairs	[192]
**(PMA)_3_InBr_6_**	35	Crystal	III	610	[InBr_6_]^3−^		LTC/WLED/STEs	[211]

## Data Availability

Not applicable.
